# Infection with pathogenic *Blastocystis* ST7 is associated with decreased bacterial diversity and altered gut microbiome profiles in diarrheal patients

**DOI:** 10.1186/s13071-022-05435-z

**Published:** 2022-09-05

**Authors:** Lei Deng, Jonathan W. J. Lee, Kevin S. W. Tan

**Affiliations:** 1 Laboratory of Molecular and Cellular Parasitology, Department of Microbiology and Immunology, Yong Loo Lin School of Medicine, National University of Singapore, 5 Science Drive 2, Singapore, 117545 Singapore; 2 Healthy Longevity Translational Research Programme, Yong Loo Lin School of Medicine, National University of Singapore, Singapore, Singapore; 3 Department of Medicine, Yong Loo Lin School of Medicine, National University of Singapore, 14 Medical Drive, Singapore, 117599 Singapore; 4 Division of Gastroenterology & Hepatology, National University Hospital, Singapore, 119074 Singapore

**Keywords:** *Blastocystis*, ST7, Gut microbiome, Bacterial diversity, Pathogenic

## Abstract

**Background:**

*Blastocystis* is a common protistan parasite inhabiting the gastrointestinal tract of humans and animals. While there are increasing reports characterizing the associations between *Blastocystis* and the gut microbiome in healthy individuals, only a few studies have investigated the relationships between *Blastocystis* and the gut microbiota in diarrheal patients.

**Methods:**

The effects of a specific subtype (ST7) of *Blastocystis* on the composition of gut microbiota in diarrheal patients were investigated using* 16S* ribosomal RNA (rRNA) gene sequencing and bioinformatic analyses.

**Results:**

Compared with diarrheal patients without *Blastocystis*, diarrheal patients infected with *Blastocystis* ST7 exhibited lower bacterial diversity. Beta diversity analysis revealed significant differences in bacterial community structure between ST7-infected and *Blastocystis*-free patients. The proportion of *Enterobacteriaceae* and *Escherichia*-*Shigella* were significantly enriched in ST7-infected patients. In contrast, the abundance of *Bacteroides* and *Parabacteroides* were more prevalent in *Blastocystis*-free patients.

**Conclusions:**

The results of this study revealed, for the first time, that infection with *Blastocystis* ST7 is associated with lower bacterial diversity and altered microbial structure in diarrheal patients. Our study on clinical diarrheal patients is also the first to reinforce the notion that ST7 is a pathogenic subtype of *Blastocystis*.

**Graphical Abstract:**

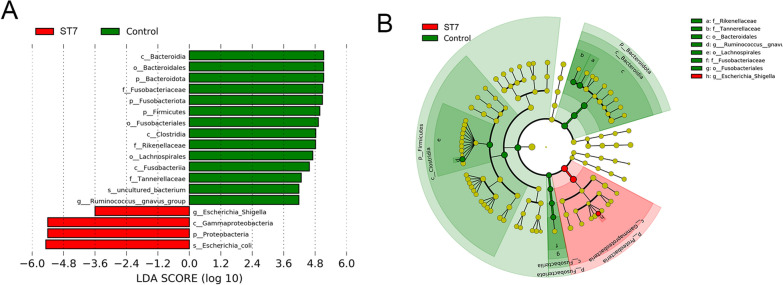

**Supplementary Information:**

The online version contains supplementary material available at 10.1186/s13071-022-05435-z.

## Background

*Blastocystis* is a genetically diverse single-celled parasite that colonizes the intestinal lumen of humans and a wide range of animals [1]. The presence of *Blastocystis* has been implicated in both asymptomatic and symptomatic hosts [2]. Clinical features attributed to *Blastocystis* include nausea, anorexia, abdominal pain, flatulence and acute or chronic diarrhea [3]. *Blastocystis* has been reported as a cause of diarrhea in the immunosuppressed population, such as renal transplant recipients [4]. It has also been suggested that *Blastocystis* is associated with irritable bowel syndrome (IBS) and inflammatory bowel disease (IBD) [5]. However, *Blastocystis* appears to be more common in healthy subjects compared to subjects with IBD, and asymptomatic *Blastocystis* carriers tend to have a higher intestinal bacterial diversity, suggesting that *Blastocystis* colonization exerts a beneficial effect on the host gut microbiota [6–8].

Whether *Blastocystis* is a pathogen or a commensal of the human gut has not yet been unequivocally determined. Given the tremendous genetic variation among *Blastocystis* subtypes, it is important to study the effect of *Blastocystis* on host health at the subtype level [9]. Several studies have shown that different subtypes exert distinct effects on host gut microbiota [8, 10]. For example, in an experimental murine model, *Blastocystis* subtype 7 (ST7) infection was found to be associated with a decrease in the beneficial bacteria of the genera *Lactobacillus* and *Bifidobacterium* [11], while infection with subtype 4 (ST4) infection promoted faster recovery from experimentally induced colitis through modulating gut bacterial compositions and immune responses [12]. The presence of subtype 3 (ST3) was positively associated with beneficial bacterial species, such as those of genera *Prevotella*, *Methanobrevibacter* and *Ruminococcus*, in patients from Italy [13].

Accumulating evidence over recent years has revealed a positive association between *Blastocystis* carriage and a diverse bacterial composition. Most of these studies focused on healthy subjects, and only a few differentiated the associations at the subtype level [9]. In our previous survey, we showed that *Blastocystis* ST7 was the predominant subtype in diarrheal patients [14], but to our knowledge no studies have investigated the relationships between ST7 infection and the gut microbiota. We performed the study reported here to better understand the associations between *Blastocystis* and gut microbiota in diarrheal patients. To this end, we characterized the gut microbiota by bioinformatic analyses of the V3-V4 region of the* 16S* ribosomal RNA (rRNA) gene in 14 *Blastocystis* ST7-infected diarrheal patients and 14 *Blastocystis*-negative diarrheal controls. Our findings provide valuable insights into the clinical significance of *Blastocystis* ST7.

## Methods

### Study population

The fecal samples used in the present study are part of a collection of fecal samples maintained at the hospital of National University of Singapore (NUH) that had been collected from patients for use in a previous study [14]. A total of 28 fecal samples (9 from males, 19 from females) were used in this study. The average age of the patients was 71 (range: 42–91) years (Additional file 1: Table S1). The study design and protocol were approved by the Domain Specific Review Board of the National Healthcare Group.

### DNA extraction and *Blastocystis* subtype identification

Genomic DNA was extracted from stool samples using the Qiagen DNA Stool Mini Kit (Qiagen, Hilden, Germany) according to the manufacturer’s instructions. The presence of *Blastocystis* was established and subtype identified as previously described [14]. Briefly, DNA samples were amplified using the primers BhRDr (5′-GAG CTT TTT AAC TGC AAC AAC G-3′) and RD5 (5′-ATC TGG TTG ATC CTG CCA GTA-3′) [15]. PCR products (around 600 bp) were subsequently cleaned up using the QIAquick® PCR Purification Kit according to the manufacturer’s instructions (Qiagen) and sent to a commercial laboratory for sequencing (Axil Scientific Pte Ltd., Singapore, Singapore). *Blastocystis* subtypes were identified by BLAST search (http://blast.ncbi.nlm.nih.gov/Blast.cgi).

### *16S* rRNA sequencing

DNA concentration was monitored using the Qubit® dsDNA HS Assay Kit (Thermo Fisher Scientific, Waltham, MA, USA). The sequencing library was constructed using a MetaVX Library Preparation Kit (Genewiz, South Plainfield, NJ, USA). Briefly, 20–30 ng of DNA was used to generate amplicons that cover the V3–V4 hypervariable regions of the* 16S* rRNA gene of bacteria. The forward primer ‘CCTACGGRRBGCASCAGKVRVGAAT’ and the reverse primer ‘GGACTACNVGGGTWTCTAATCC’ were used. PCR cycling was performed in a reaction volume of 25 µl containing 2.5 µl of TransStart buffer, 2 µl of dNTPs, 1 µl of each primer, 0.5 µl of TransStart Taq DNA polymerase and 20 ng template DNA. The cycling conditions were: 3 min of denaturation at 94 °C, following by 5 s at 95 °C, 90 s of annealing at 57 °C and 10 s of elongation at 72 °C, for 24 cycles, with a final extension at 72 °C for 5 min. Indexed adapters were added to the ends of the amplicons by limited cycle PCR. Finally, the library was purified with magnetic beads. The concentration was determined using a microplate reader (Infinite 200 Pro; Tecan Group Ltd., Männedorf, Switzerland), and the expected fragment size of  approximately 600 bp was confirmed by 1.5% agarose gel electrophoresis. Next-generation sequencing was conducted on an Illumina Novaseq Platform (Illumina, Inc., San Diego, CA, USA) at the laboratory. Automated cluster generation and 250 paired-end sequencing with dual reads were performed according to the manufacturer’s instructions.

### Bioinformatic and microbiota diversity analyses

Paired-end sequencing of positive and negative reads were filtered, followed by denoising and chimera removal using the QIIME2 DADA2 plug-in to obtain amplicon sequence variants (ASVs) [16]. Taxonomic classifications were assigned to the ASV table using the Ribosomal Database Project (RDP) Classifier, which is a Bayes algorithm-fitted classifier trained on the Silva v138 database [17]. Based on the results of ASV analysis, an alpha diversity index, species richness and species evenness can be derived for each sample, with the Shannon and Simpson indices used to reflect bacterial richness and evenness, respectively. Observed ASVs and the Chao1 richness estimate were used to estimate bacterial richness. Principal co-ordinates analysis (PCoA) plots were constructed based on Bray–Curtis dissimilarity to illustrate the differences in community structure between the different groups. Heatmaps were used to show the different taxa between groups. Linear discriminant analysis effect size (LEfSe) analysis was performed to detect bacterial taxa with significantly different abundance among different groups with *P*-value < 0.05 and linear discriminant analysis (LDA) score > 2 (https://huttenhower.sph.harvard.edu/galaxy/). Bacterial differential abundance analysis was also carried out with ALDEx2 in R using center log-transformed data [18].

### Statistical analysis

Statistical analysis was performed using R-4.0.3 software (R Foundation for Statistical Computing, Vienna, Austria) and GraphPad Prism 8 software (GraphPad Software Inc., San Diego, CA, USA). Significant differences in alpha diversity between groups were determined using a Mann–Whitney–Wilcoxon (MWW) test, differences in beta diversity were tested by permutational multivariate analysis of variance (PERMANOVA) and significant differences in relative abundance were assessed using the Wilcoxon rank-sum test.

## Results

### Assessment of sequence data

High-throughput Illumina Novaseq sequencing of the* 16S* rRNA gene generated a total of 4,557,937 paired reads, of which 3,701,427 high-quality reads were selected for the bioinformatic analysis. The average number of reads per sample was 132,193 ± 12,066. The rarefaction curve was used to reflect the sequencing depth and indirectly evaluate species richness from the sampling results. Our data showed that sufficient sequencing depth was obtained and that ST7-infected patients seemed to have a lower number of ASVs than uninfected patients (Additional file 2: Figure S1).

### *Blastocystis* ST7 infection is associated with decreased alpha diversity of gut bacterial microbiota

**Fig. 1:**
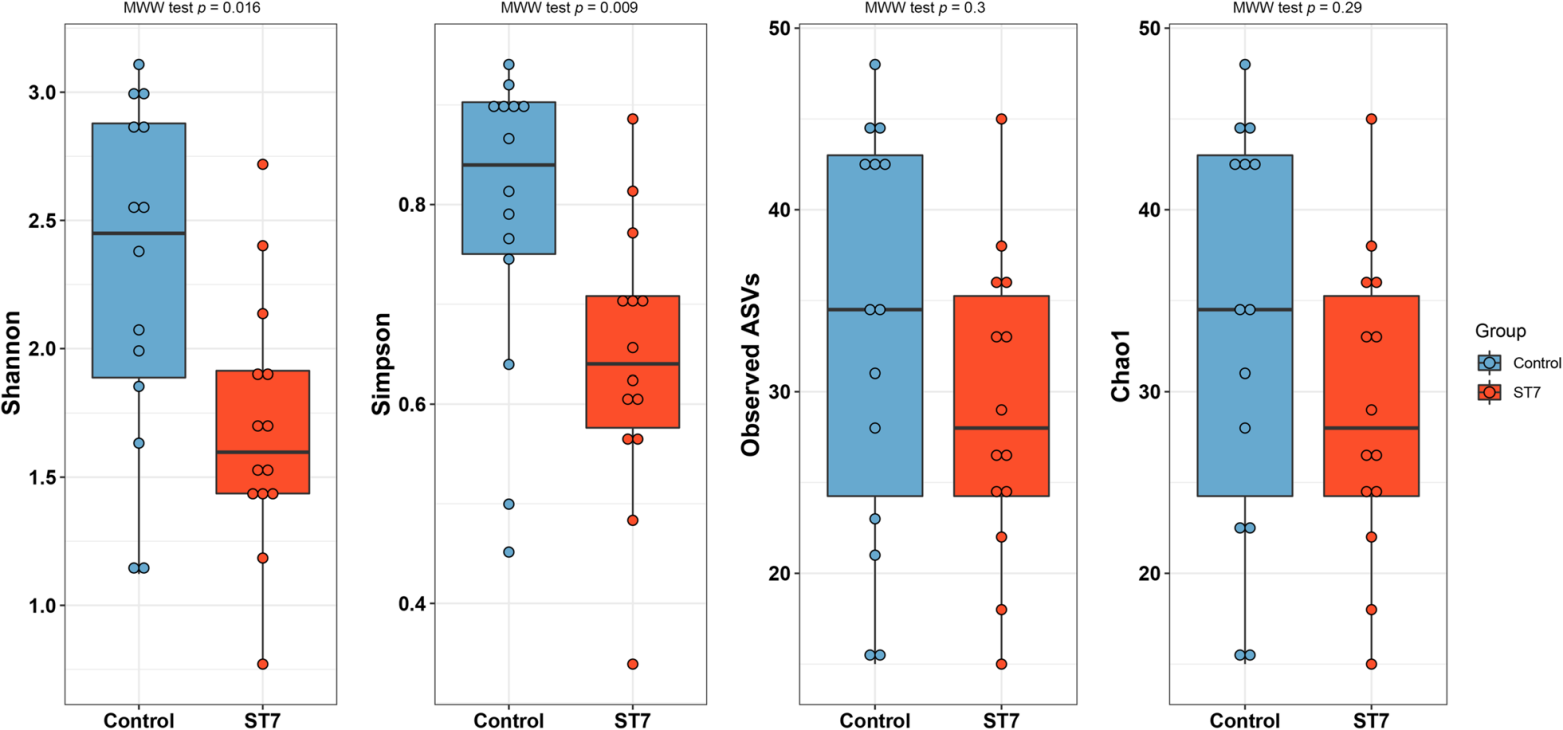
Box plots of the Shannon, Simpson and Chao1 indices and observed ASVs in *Blastocystis* ST7-infected patients and the non-*Blastocystis* controls, compared using the Mann–Whitney-Wilcoxon test. Abbreviations: ASV, Amplicon sequence variants; ST, subtype

Four indices, including the Shannon, Simpson and Chao1 indices and observed ASVs, were used to reflect the alpha diversity of the gut bacterial community. Both the Shannon and Simpson diversity indices, which reflect the bacterial richness and evenness, respectively, showed that the diversity of the gut bacterial communities in ST7-infected patients was lower than that in *Blastocystis* non-infected subjects (MWW test, *P* < 0.05) (Fig. 1). Observed ASVs and Chao1 indices were used to reflect the richness of the gut bacterial communities. Although ST7-infected patients showed lower bacterial richness, the difference did not reach significance when compared to *Blastocystis* non-infected subjects (MWW test, *P* > 0.05) (Fig. 1).

### Relationship between *Blastocystis* ST7 infection and the gut microbiome beta diversity

**Fig. 2:**
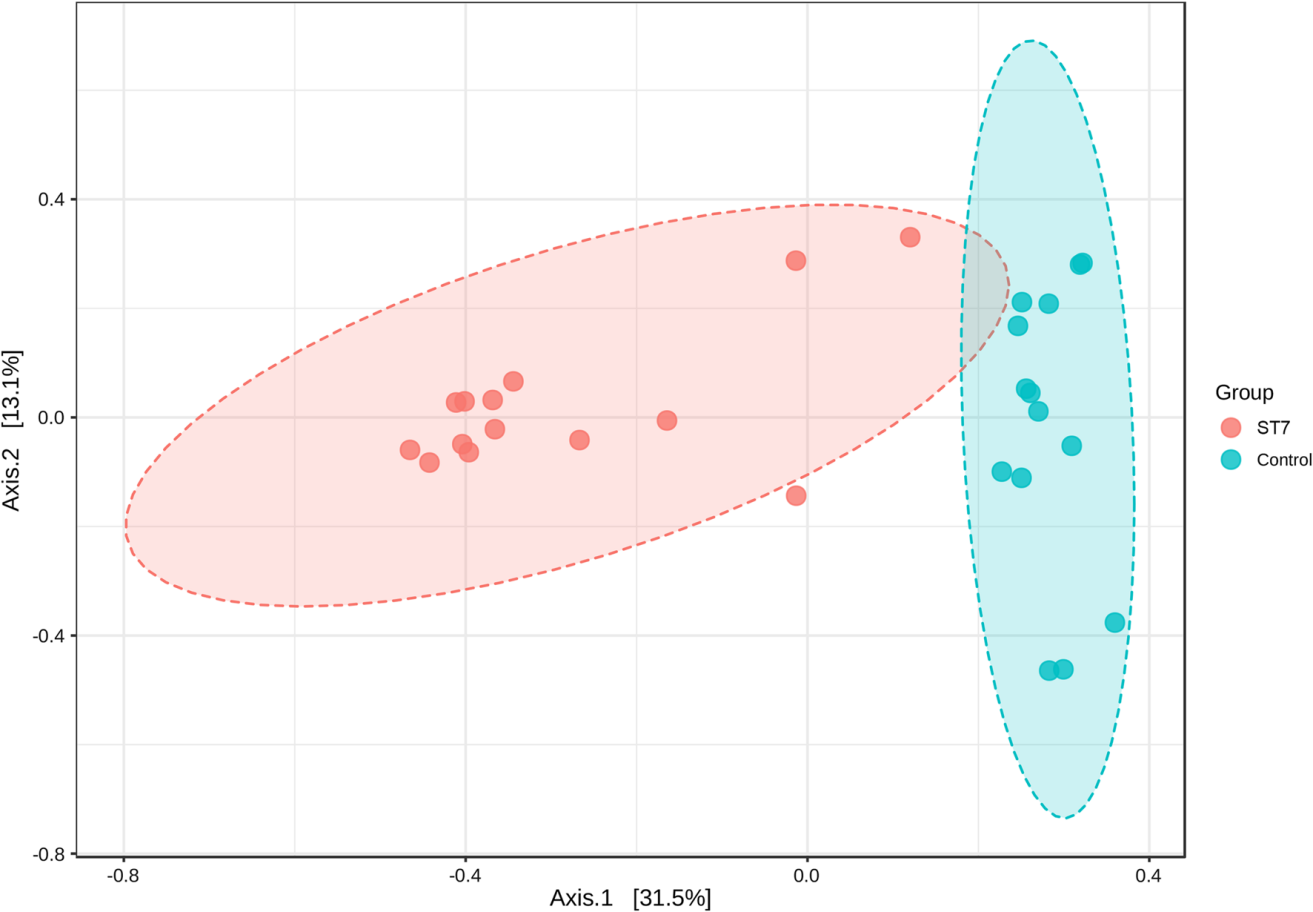
Composition of gut microbiota was significantly altered in *Blastocystis* ST7-infected patients. Microbial composition is represented by beta diversity based on Bray–Curtis dissimilarity distance

The Bray–Curtis dissimilarity index, which was used to assess the differences in bacterial community structure between *Blastocystis* ST7-infected and non-infected patients, was analyzed using PCoA and PERMANOVA. The PCoA plot showed a clustering of the samples that depended on the *Blastocystis* ST7 infection status of the patients; PC coordinate 1 (31.5%) and PC coordinate 2 (13.1%) scores explained 44.6% of the variance of the data (Fig. 2). PERMANOVA analysis showed a statistically significant difference in the bacterial community structure between the infected and non-infected groups (*F*-value = 9.4615, *R*^2^ = 0.26681, *P* < 0.001).

### Impact of *Blastocystis* ST7 infection on gut bacterial communities

**Fig. 3:**
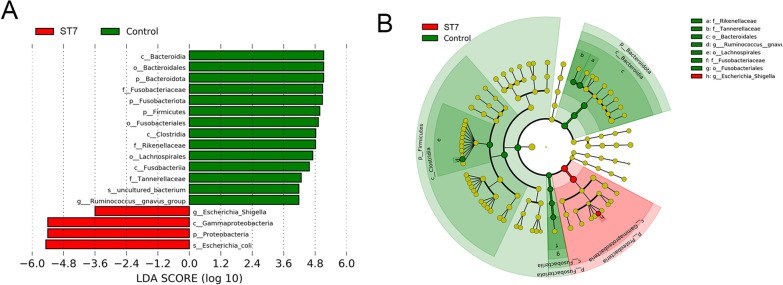
**a** Taxa with significantly differences in different groups were detected by LEfSe analysis. **b** Cladogram generated by LEfSe indicating differentially abundant bacterial taxa. Abbreviations: LDA, Linear discriminant analysis; LEfSe, linear discriminant analysis effect size

**Fig. 4:**
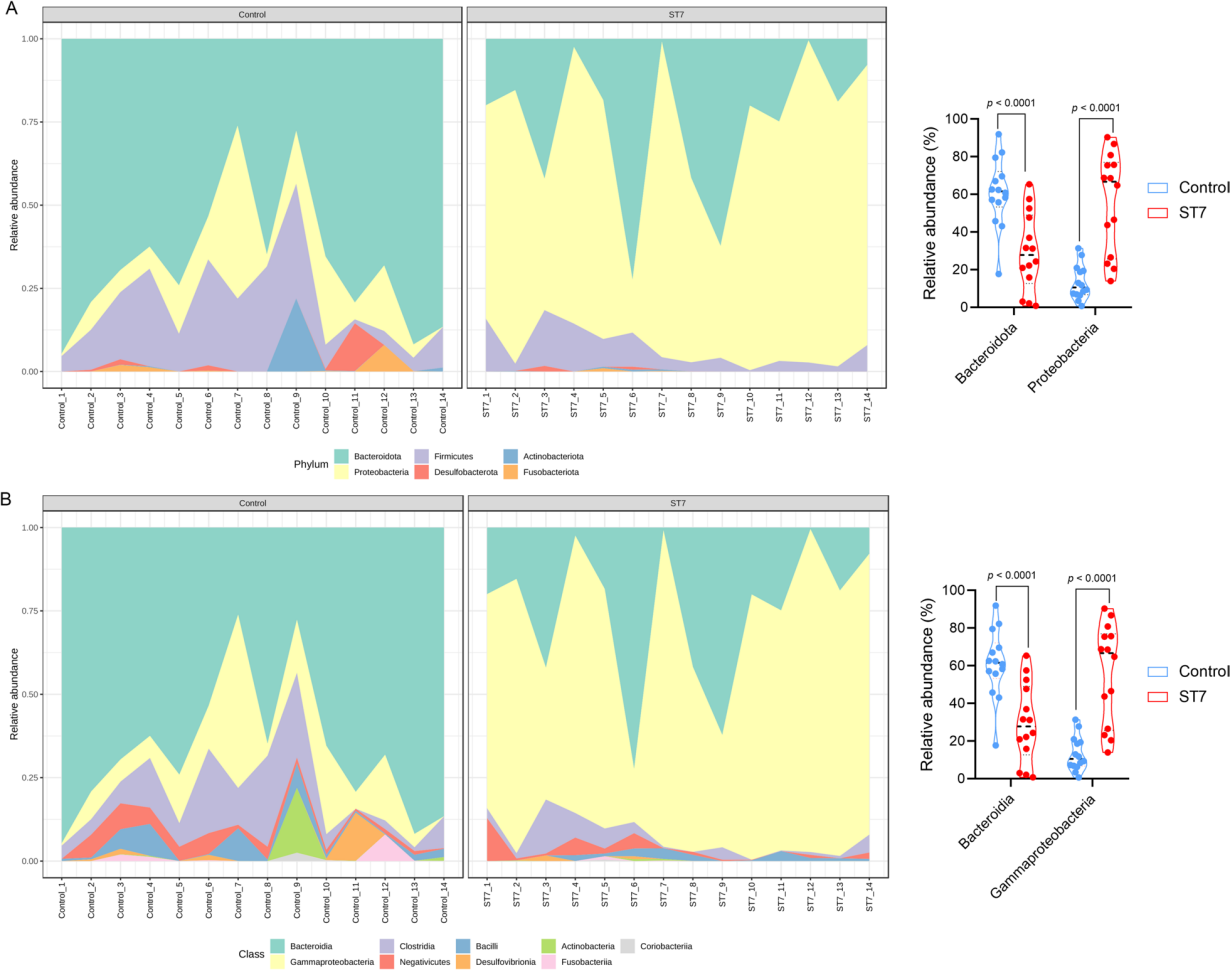
**a** Distribution of the gut microbiota by phylum in *Blastocystis* ST7-infected patients and non-*Blastocystis* controls (left). Relative abundances of the two different phyla between two groups (right). **b** Distribution of the gut microbiota according to class in *Blastocystis* ST7-infected patients and non-*Blastocystis* controls (left). Relative abundances of the two different classes between the two groups (right). Analysis was performed using the Wilcoxon rank-sum test

The LEfSe analysis was used to identify those bacterial taxa which were present at a significantly different abundance in *Blastocystis* ST7-infected and non-infected patients. *Proteobacteria* (phylum), *Gammaproteobacteria* (class), *Escherichia-Shigella* (genus) and *Escherichia coli* (species) were found to be enriched in ST7-infected patients (Fig. 3a, b). Similarly, bacterial compositional analysis showed that the phylum *Proteobacteria* was significantly enriched in ST7-infected patients when compared to *Blastocystis* non-infected patients, while the phylum *Bacteroidota* was less represented in ST7-infected patients (Wilcoxon test, *P* < 0.0001; Fig. 4a). At the class level, we observed a higher abundance of *Gammaproteobacteria* and a lower level of *Bacteroidia* in ST7-infected patients (Wilcoxon test, *P* < 0.0001; Fig. 4b). Regarding the distribution of bacterial orders, *Bacteroidales* was enriched in *Blastocystis*-free patients, and a higher proportion of *Enterobacterales* was observed in ST7-infected subjects (Wilcoxon test, *P* < 0.0001, Additional file 3: Figure S2). Similarly, a higher trend of *Enterobacteriaceae* was observed in ST7-infected patients, whereas the proportion of *Bacteroidaceae* was lower in the ST7-infected patients (Wilcoxon test, *P* < 0.0001; Additional file 3: Figure S2). The heatmap shows the relative abundances of genera in fecal microbiota between the ST7-infected and non-infected groups at the genus level (Additional file 4: Figure S3). The proportion of *Escherichia-Shigella* was higher in patients infected with *Blastocystis* ST7 than in *Blastocystis*-free individuals (Additional file 4: Figure S3). In contrast, the levels of *Bacteroides* and *Parabacteroides* were enriched in *Blastocystis*-free patients (Additional file 4: Figure S3). Differential abundance analysis of bacterial ASVs using the ALDEx2 package revealed that *Blastocystis* ST7 infection was significantly correlated with four bacterial taxa, namely *Escherichia-Shigella*, *Escherichia-Shigella* unclassified, *Escherichia coli* and *Phascolarctobacterium* (Additional file 5: Figure S4).

## Discussion

Although numerous early studies reported that *Blastocystis* was associated with acute or chronic digestive disorders, its clinical significance and pathogenicity remain unclear. *Blastocystis* can be found in both asymptomatic and symptomatic patients, while only a few studies have investigated the etiological role of *Blastocystis* in gastrointestinal diseases. Our study is the first to investigate and report the effects of the rare *Blastocystis* ST7 on gut bacterial communities in patients with gastrointestinal disorders.

The pathogenicity of ST7 has been determined in both in vitro and in vivo experiments [9, 19]. For example, ST7 infection induced the degradation of immunoglobulin A (IgA) and disrupted the epithelial barrier in colonic epithelial cell lines [20, 21]. ST7 infection also increased the release of pro-inflammatory cytokines, such as interleukin (IL)-6, IL-1β and tumor necrosis factor alpha (TNFα), in murine macrophages mediated by mitogen-activated protein kinases (MAPKs) and enhanced the effect of lipopolysaccharide (LPS)-mediated NF-κB pathway activation [22, 23]. Furthermore, ST7 infection also caused colonic pathology changes in a mouse model [11]. Our previous study showed that ST7 was the predominant subtype in diarrheal patients, indicating its potential pathogenicity to humans [14]. The present study further characterized the association of ST7 infection on the gut microbiome in diarrheal patients. We observed that ST7-infected patients showed decreased bacterial diversity and a higher abundance of the ‘harmful’ bacteria *Escherichia-Shigella*.

Several studies have investigated the associations between *Blastocystis* colonization and diversity of the gut bacterial microbiota [7, 10, 24–27]. Microbial diversity is considered to be a potential biomarker of a healthy gut, and higher diversity means stability and resilience of the gut ecosystem [28]. A reduction in intestinal microbiota diversity has been observed in a series of digestive diseases, such as IBD and IBS, that are associated with inflammation of the lower gastrointestinal tract [29, 30]. Most of the studies conducted to date have shown that *Blastocystis* colonization is associated with a higher bacterial diversity of gut microbial communities [25–27]. However, in contrast, in the present study we found that the presence of *Blastocystis* ST7 was associated with a lower bacterial diversity. There is a dearth of information on *Blastocystis* ST7 and the gut microbiome of humans because ST7 is a relatively rare subtype in humans, and most microbiome studies are based on healthy individuals, while ST7 is usually reported in symptomatic individuals [14, 31].

Previous studies also showed that carriers of *Blastocystis* had a higher abundance of *Firmicutes* and a lower abundance of *Proteobacteria*, compared to *Blastocystis*-free individuals [27, 32]. At the phylum level, *Proteobacteria* has a low abundance in the gut of healthy humans, and the expansion of *Proteobacteria* is usually associated with a compromised ability to maintain a balanced gut microbial community which, in turn, is a potential diagnostic signature of dysbiosis and risk of disease [33]. In addition, the *Escherichia*-*Shigella* group, belonging to phylum *Proteobacteria* and family *Enterobacteriaceae*, is one of the most important group of enteric pathogens causing gastroenteritis worldwide [34]. It has been determined that *Enterobacteriaceae* abundance correlates positively with gastrointestinal or systemic inflammation [35], and a higher abundance of *Enterobacteriaceae* is commonly observed in patients with IBD [36]. *Blastocystis*-colonized patients were found to exhibit a lower proportion of *Enterobacteriaceae* in different studies [7, 26], suggesting that *Blastocystis* colonization is associated with healthy gut microbiota. Surprisingly, our data showed a greater abundance of the *Enterobacteriaceae* in ST7-infected patients, suggesting that ST7 infection is associated with intestinal inflammation. These results also highlight the importance of *Blastocystis* subtyping in microbiota studies.

The associations between *Blastocystis* and *Bacteroides* have been elaborated in several studies [8, 32, 37, 38]. Species of genus *Bacteroides* are normally mutualistic or commensal and constitute the most important part of the mammalian gastrointestinal microbiota [39]. They play a complex role in the processing of energy absorption, carbohydrate degradation and host intestinal health [39, 40]. Some studies have implicated *Bacteroides* in the development of chronic inflammation of the gastrointestinal tract [41, 42], while *Bacteroides* are also known as the primary producers of short-chain fatty acids in the human gut, and are crucial in maintaining the stability of the immune system [43, 44]. In healthy individuals, *Blastocystis* colonization has been reported to decrease the proportion of *Bacteroides* [37] and to be less prevalent in *Bacteroides* enterotype samples [8]. Similarly, the role of *Parabacteroides* on host health is also complicated. The enrichment of *Parabacteroides* species was found to be strongly correlated with disease activity in a patient with ulcerative colitis [45], while it also determined that species from *Parabacteroides* can alleviate *E. coli* LPS-induced IL-8 production in vitro, suggesting it may exhibit anti-inflammatory capacity [46]. Interestingly, our data also showed that ST7 infection decreased the abundance of *Bacteroides* and *Parabacteroides* species in diarrheal patients, but the etiological role of *Bacteroides* and *Parabacteroides* in intestinal inflammation needs more clarification in future studies.

It is worth noting that the present study only shows data on ST7 and its association with microbial diversity in diarrheal patients; data on the association of other subtypes with diarrheal patients remain limited. Another confounding factor is that some of the diarrheal patients tested positive for *Clostridioides difficile*, which is a potential factor contributing to an imbalance in the microbiota; however, we did not differentiate its effects on gut microbial communities with *Blastocystis* ST7 due to the small number of patients included in the present study. Future studies should include more samples and subtypes to compare the effects of a specific subtype on gut microbial community and host health.

## Conclusion

The current study reported, for the first time, that *Blastocystis* ST7 infection was associated with lower bacterial diversity and richness of gut bacterial communities in diarrheal patients. In addition, *Blastocystis* ST7-infected patients showed a higher proportion of ‘harmful’ bacteria (*Proteobacteria*) in the human gut. Our data suggest that *Blastocystis* ST7 may interact with multiple members of the microbiota, such as the *Escherichia*-*Shigella* group, to cause these negative alterations. These findings highlight the importance of stratifying *Blastocystis* infections into subtypes and may provide new guidelines for the treatment of clinical blastocystosis.

## Supplementary Information

**Additional file 1: Table S1.** Samples used in the present study.

**Additional file 2: Figure S1.** Rarefaction curves. The* x*-axis shows the number of reads per sample and the* y*-axis shows the number of ASVs. Each curve in the graph represents a different sample and the samples in the same group are represented by a uniform color. ASVs: amplicon sequence variants.

**Additional file 3: Figure S2. a** The order distribution of the gut microbiota of *Blastocystis* ST7-infected patients and non-*Blastocystis* controls (left). Relative abundances of the two different orders between two groups (right). **b** The distribution of gut microbiota of *Blastocystis* ST7-infected patients and non-*Blastocystis* controls (left), according to family. Relative abundances of the two different families between two groups (right). Wilcoxon rank-sum test.

**Additional file 4: Figure S3.** Heatmap of ST7 infection-associated taxonomic markers.

**Additional file 5: Figure S4.** Association of gut bacterial composition with the presence of *Blastocystis* ST7. ASV fold change versus median abundance (left), and ASV fold change between versus within conditions (right). Red markings indicate ASVs with significant changes (Wilcoxon rank test).

## Data Availability

The raw sequence data from the fecal microbiota in this paper were uploaded to the Sequence Read Archive (SRA) database at NCBI under BioProject ID PRJNA850541 (https://www.ncbi.nlm.nih.gov/bioproject/PRJNA850541).

## Abbreviations

ASVs: Amplicon sequence variants
IBD: Inflammatory bowel disease
IBS: Irritable bowel syndrome
IgA: Immunoglobulin A
LEfSe: Linear discriminant analysis effect size
MAPKs: Mitogen-activated protein kinases
MWW: Mann–Whitney–Wilcoxon
NUH: National University Hospital
PCoA: Principal co-ordinates analysis
PERMANOVA: Permutational multivariate analysis of variance
RDP: Ribosomal database program
ST: Subtype
