# Supplementary material for: Infection with pathogenic Blastocystis ST7 is associated with decreased bacterial diversity and altered gut microbiome profiles in diarrheal patients
Source: Parasit Vectors. 2022 Sep 5;15:312. doi: 10.1186/s13071-022-05435-z (PMC9446694; doi:10.1186/s13071-022-05435-z)
Supplement: Supplementary file 1 — Additional file 1: Table S1. Samples used in the present study. [file 13071_2022_5435_MOESM1_ESM.docx]

**Table S1. The samples used in the present study.**

| **Sample ID** | **Gender** | **Age** | **Group** |
| --- | --- | --- | --- |
| N18 | Female | 61Y | Control_1 |
| N46 | Female | 73Y | Control_2 |
| N47 | Female | 59Y | Control_3 |
| N50 | Female | 69Y | Control_4 |
| N83 | Female | 64Y | Control_5 |
| N86 | Male | 67Y | Control_6 |
| N87 | Male | 65Y | Control_7 |
| A33 | Female | 79y | Control_8 |
| A34 | Female | 70y | Control_9 |
| A36 | Female | 84y | Control_10 |
| A39 | Female | 87y | Control_11 |
| A48 | Female | 91y | Control_12 |
| A49 | Female | 80y | Control_13 |
| A50 | Male | 83y | Control_14 |
| N31 | Male | 53Y | ST7_1 |
| N32 | Female | 55Y | ST7_2 |
| N33 | Female | 76Y | ST7_3 |
| N37 | Male | 79Y | ST7_4 |
| N39 | Male | 42Y | ST7_5 |
| N40 | Female | 90Y | ST7_6 |
| N42 | Male | 65Y | ST7_7 |
| N44 | Female | 88y | ST7_8 |
| N92 | Male | 63Y | ST7_9 |
| A1 | Male | 65Y | ST7_10 |
| A2 | Female | 91Y | ST7_11 |
| A3 | Female | 56Y | ST7_12 |
| A4 | Female | 63Y | ST7_13 |
| A8 | Female | 71Y | ST7_14 |
