# Supplementary figures and images for: Infection with pathogenic Blastocystis ST7 is associated with decreased bacterial diversity and altered gut microbiome profiles in diarrheal patients
Source: Parasit Vectors. 2022 Sep 5;15:312. doi: 10.1186/s13071-022-05435-z (PMC9446694; doi:10.1186/s13071-022-05435-z)

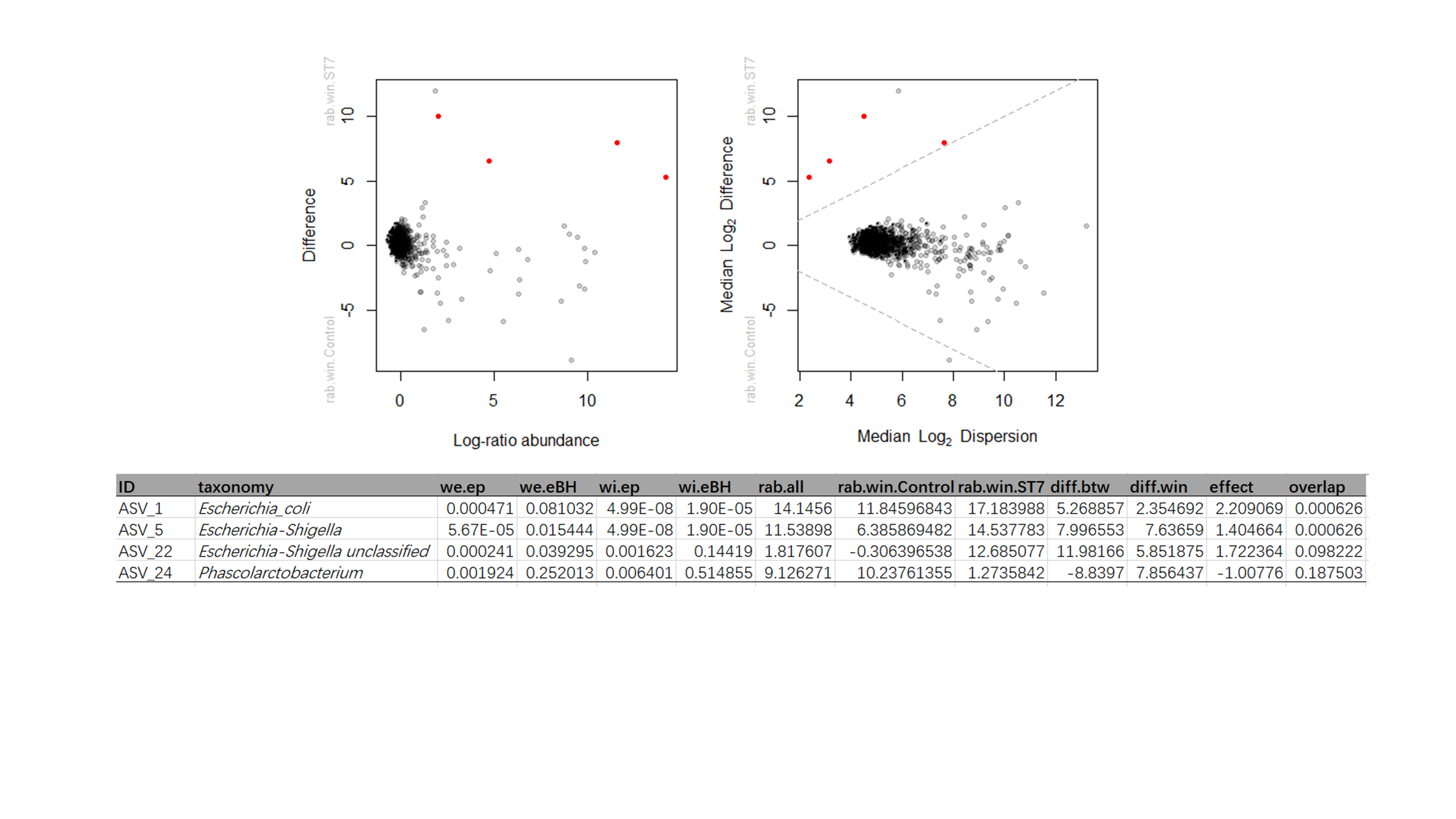

Supplement: Supplementary file 2 — Additional file 2: Figure S1. Rarefaction curves. The x-axis shows the number of reads per sample and the y-axis shows the number of ASVs. Each curve in the graph represents a different sample and the samples in the same group are represented by a uniform color. ASVs: amplicon sequence variants. [file 13071_2022_5435_MOESM2_ESM.tif]

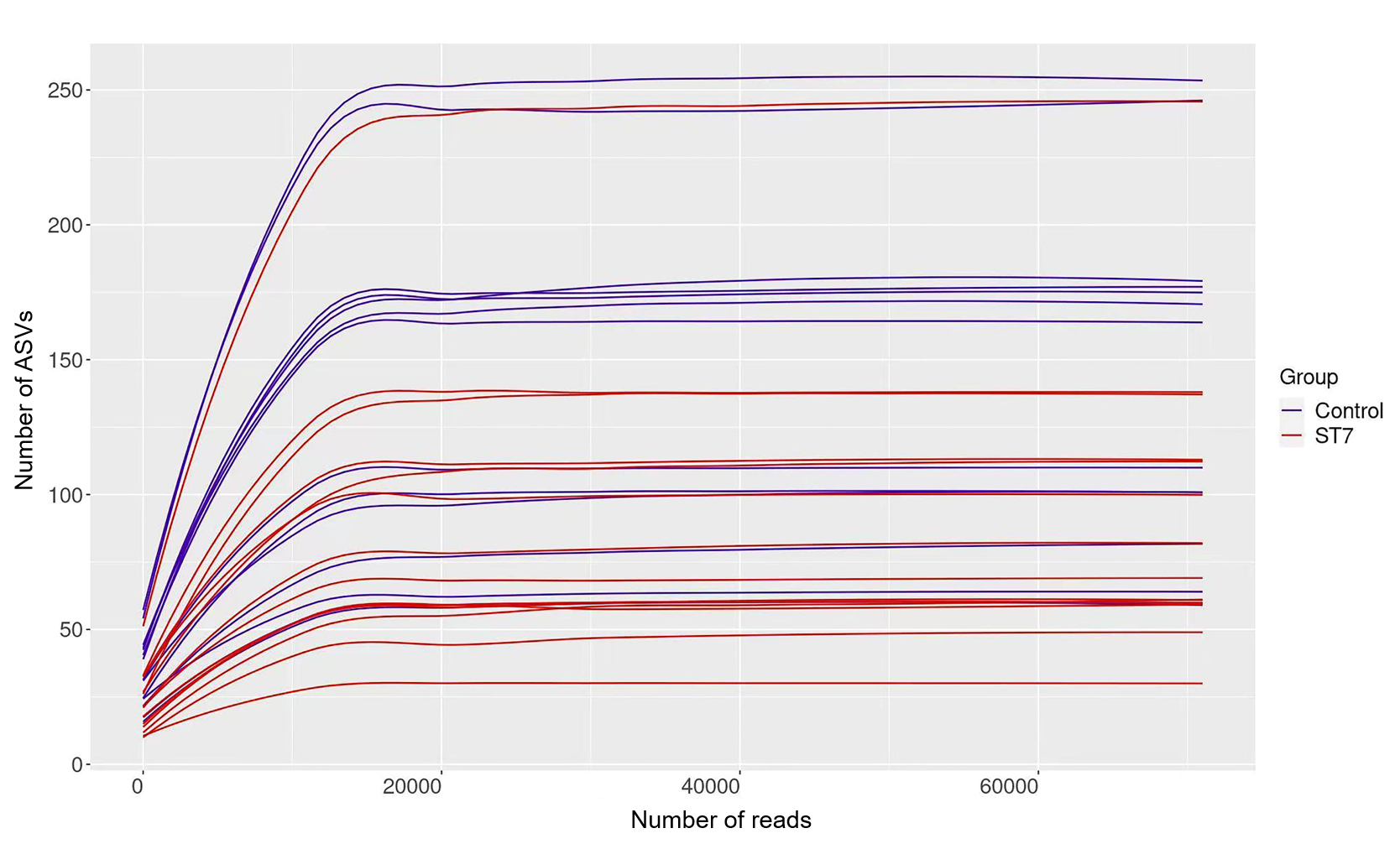

Supplement: Supplementary file 3 — Additional file 3: Figure S2. a The order distribution of the gut microbiota of Blastocystis ST7-infected patients and non-Blastocystis controls (left). Relative abundances of the two different orders between two groups (right). b The distribution of gut microbiota of Blastocystis ST7-infected patients and non-Blastocystis controls (left), according to family. Relative abundances of the two different families between two groups (right). Wilcoxon rank-sum test. [file 13071_2022_5435_MOESM3_ESM.tif]

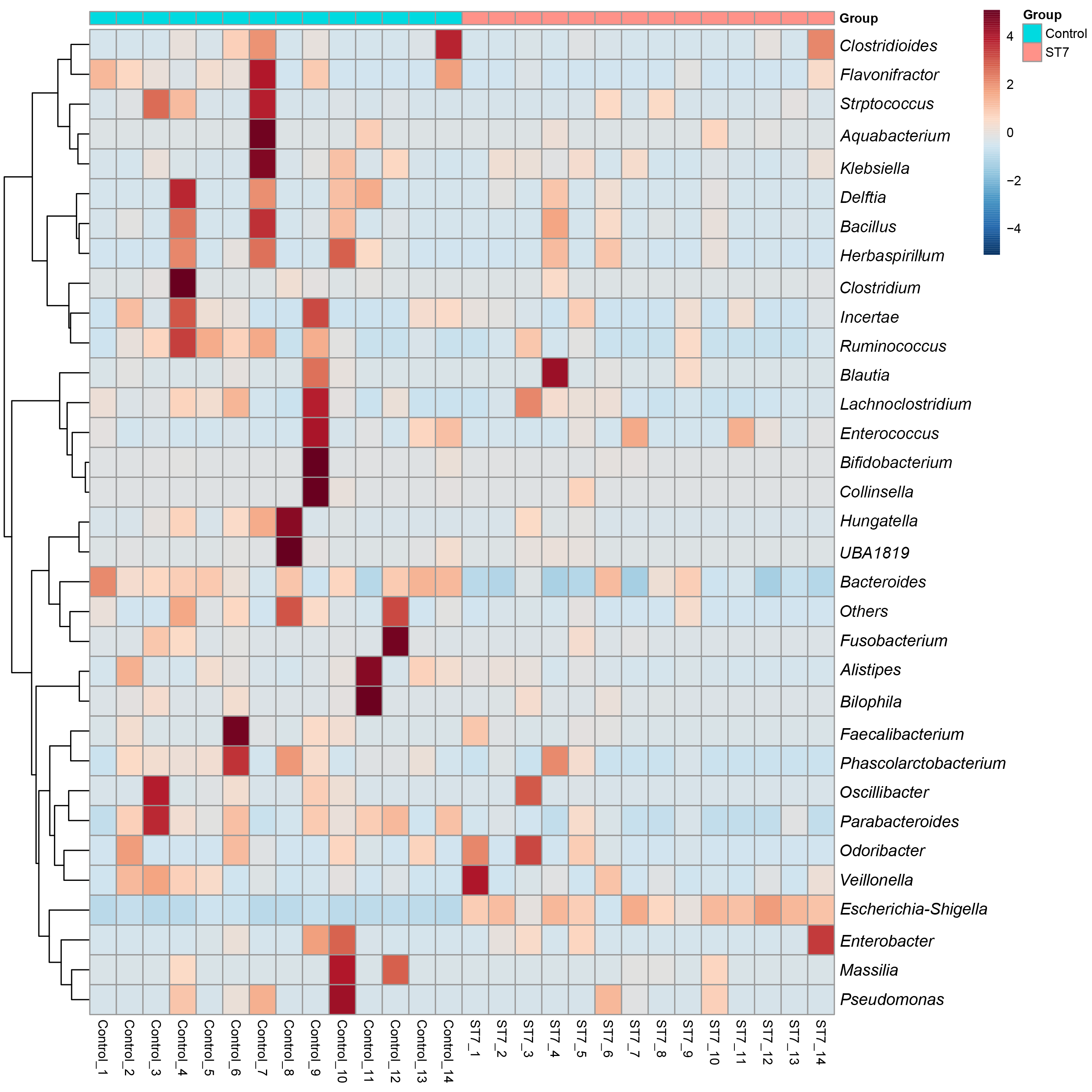

Supplement: Supplementary file 4 — Additional file 4: Figure S3. Heatmap of ST7 infection-associated taxonomic markers. [file 13071_2022_5435_MOESM4_ESM.tif]

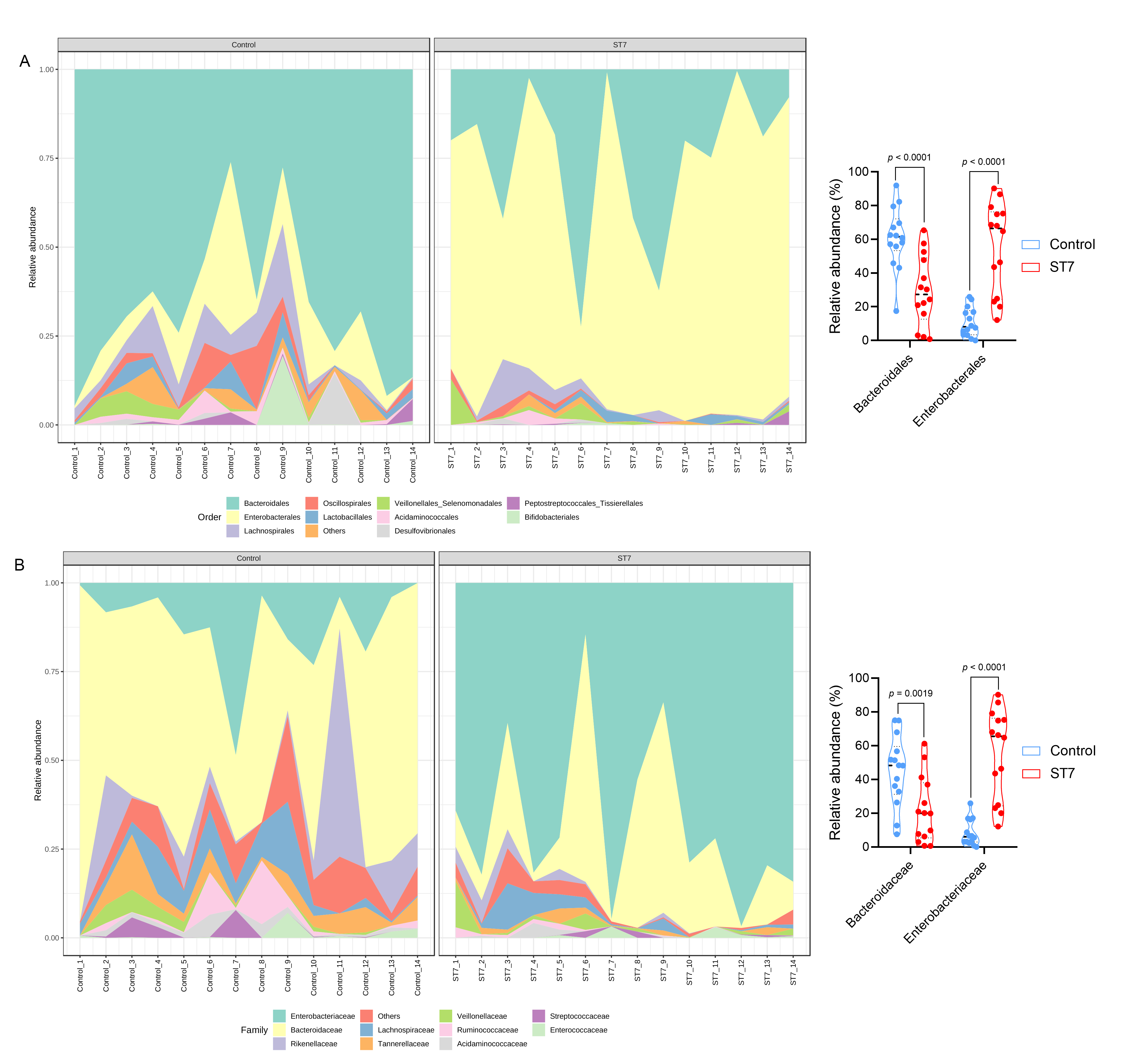

Supplement: Supplementary file 5 — Additional file 5: Figure S4. Association of gut bacterial composition with the presence of Blastocystis ST7. ASV fold change versus median abundance (left), and ASV fold change between versus within conditions (right). Red markings indicate ASVs with significant changes (Wilcoxon rank test). [file 13071_2022_5435_MOESM5_ESM.tif]
